# The Management of Oral Pemphigus Vulgaris in a Hypertensive Patient: A Case Report

**DOI:** 10.7759/cureus.48184

**Published:** 2023-11-02

**Authors:** Rosalyn Lalremtluangi, Suwarna Dangore-Khasbage, Swapnil Mohod

**Affiliations:** 1 Oral Medicine and Radiology, Sharad Pawar Dental College and Hospital, Datta Meghe Institute of Higher Education and Research, Wardha, IND

**Keywords:** steroids, pemphigus vulgaris, management, lesion, hypertension

## Abstract

Pemphigus vulgaris (PV) is a chronic autoimmune disorder that causes painful blisters on the skin and mucosa along with erosions due to intra-epithelial acantholysis. This acantholysis is mainly due to an immune reaction against desmoglein (an adhesion glycoprotein molecule) by IgG autoantibodies, which causes loss of cell-to-cell adhesion. The treatment consists of systemic corticosteroids such as tablet prednisolone along with topical steroids such as 0.1% triamcinolone paste. But steroids are known for their side effects, one of which is hypertension. It is vital for a dentist to curate the management of this lesion particularly when the drug of choice can potentially lead to adverse effects including systemic complications. This is a case report of a 60-year-old female with PV with systemic hypertension, emphasizing the management of this condition so as to prevent any complications that may arise due to the drug that is administered.

## Introduction

Pemphigus vulgaris (PV) is a long-standing autoimmune disorder that is manifested on the skin and mucosa as painful blisters and erosions, which are caused by intra-epithelial acantholysis [1]. The immune reaction against glycoproteins desmoglein 1 and 3 (an adhesion molecule) by IgG autoantibodies causes the loss of adhesion of one cell to another cell, which can lead to blisters in the intra-epithelial region [2]. These desmosomes help in the cell-cell junction of the epidermis by attaching the keratin intermediate filament to the epidermal cell membrane [3]. The “desmoglein compensation theory” and “multiple hits hypothesis” are the two among several proposed concepts, which explain the pathophysiology of pemphigus [3].

Initially, a thin-walled bulla arises on normal-looking mucosa or skin. Then, the bulla breaks rapidly and continues to spread peripherally and eventually leaves a large denuded area on the skin [4]. When pressure is applied to apparently normal skin, it may end up in the formation of a new lesion, which is called Nikolsky’s sign, resulting from pulling away the upper layer of the skin from the basal layer. The diagnosis of PV is often aided by clinical presentation with long long-standing history of ulceration, direct immunofluorescence microscopy, serology, or lesional biopsy [5]. The standard treatment used for PV up to this day is systemic and topical steroids, which mainly aid in reducing symptoms and controlling the acute phase with high dosage and tapering the dose as maintenance therapy. Although steroids are strong anti-inflammatory agents and have successfully treated certain inflammatory lesions and conditions, they have several adverse effects and complications, one of which is hypertension [5]. Due to this very reason, it may be controversial to administer systemic steroids to patients who are hypertensive as it may cause a more serious complication, even leading to hypertensive crisis [6]. As the patient was hypertensive and one of the side effects of systemic steroid is rise in blood pressure, the management was challenging. This is a case presentation of a 60-year-old female patient who was having PV with hypertension. This article emphasizes on the management of PV with hypertensive patient and measures to prevent systemic complication.

## Case presentation

A 60-year-old female came to the Department of Oral Medicine and Radiology at Sharad Pawar Dental College and Hospital, Datta Meghe Institute of Higher Education and Research (DMIHER), with a chief complaint of multiple ulcerations in the oral cavity for three months. History revealed that the patient first experienced irritation and burning sensation in the oral cavity three and half months back, and after approximately seven days, she noticed clusters of tiny ulcers in both cheeks, which coalesced with each other after a few days. These ulcers extended toward the posterior region of the mouth and later involved both sides of the tongue. The patient also reported difficulty in mastication and swallowing and an increase in salivation for 20 days. The patient has not taken any medication for her chief complaint. The patient also gave a history of hypertension for one year and was on regular medication (tablet enalapril maleate 5 mg once daily) for the same. All her vital signs were within normal limits, except her blood pressure that is above normal value (160/90 mm Hg).

**Figure 1:**
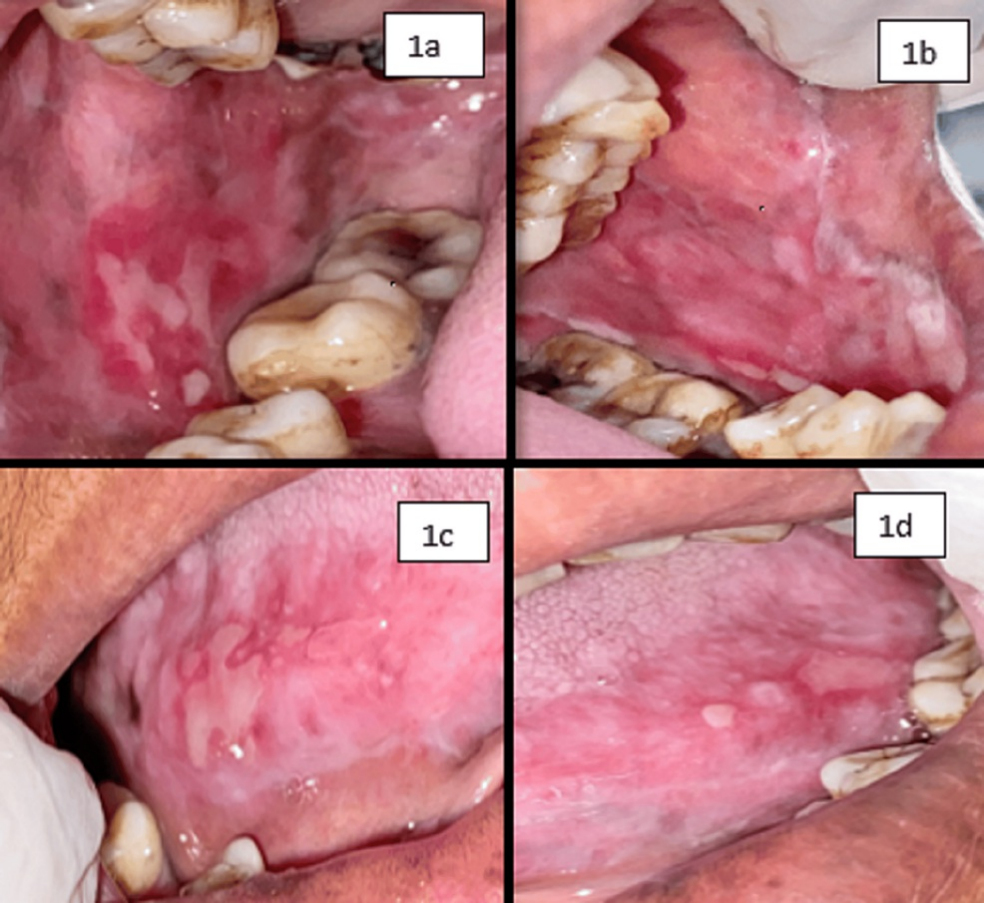
Intraoral lesion of the patient on the first visit. Figure 1a shows the right buccal mucosa, Figure 1b shows the left buccal mucosa, Figure 1c shows the right lateral border of the tongue, and Figure 1d shows the left lateral border of the tongue.

**Figure 2:**
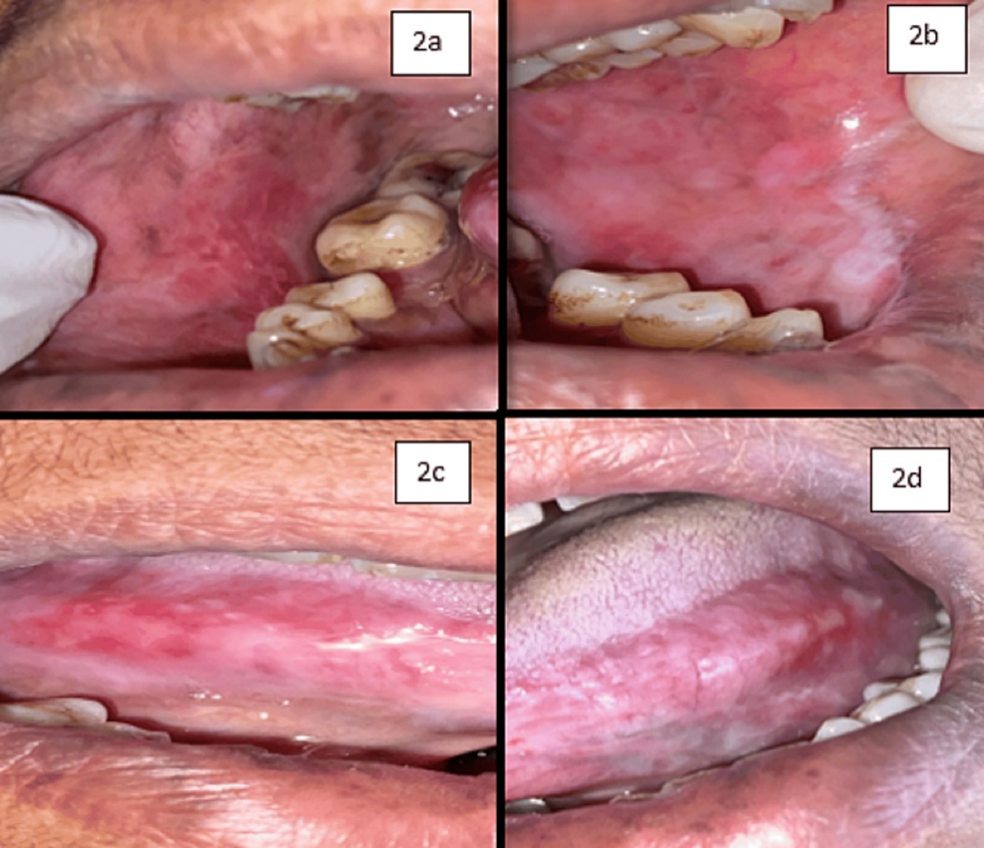
Second visit where the healing of lesions was evident (50%-60%). Figure 2a shows the right buccal mucosa, Figure 2b shows the left buccal mucosa, Figure 2c shows the right lateral border of the tongue, and Figure 2d shows the left lateral border of the tongue.

**Figure 3:**
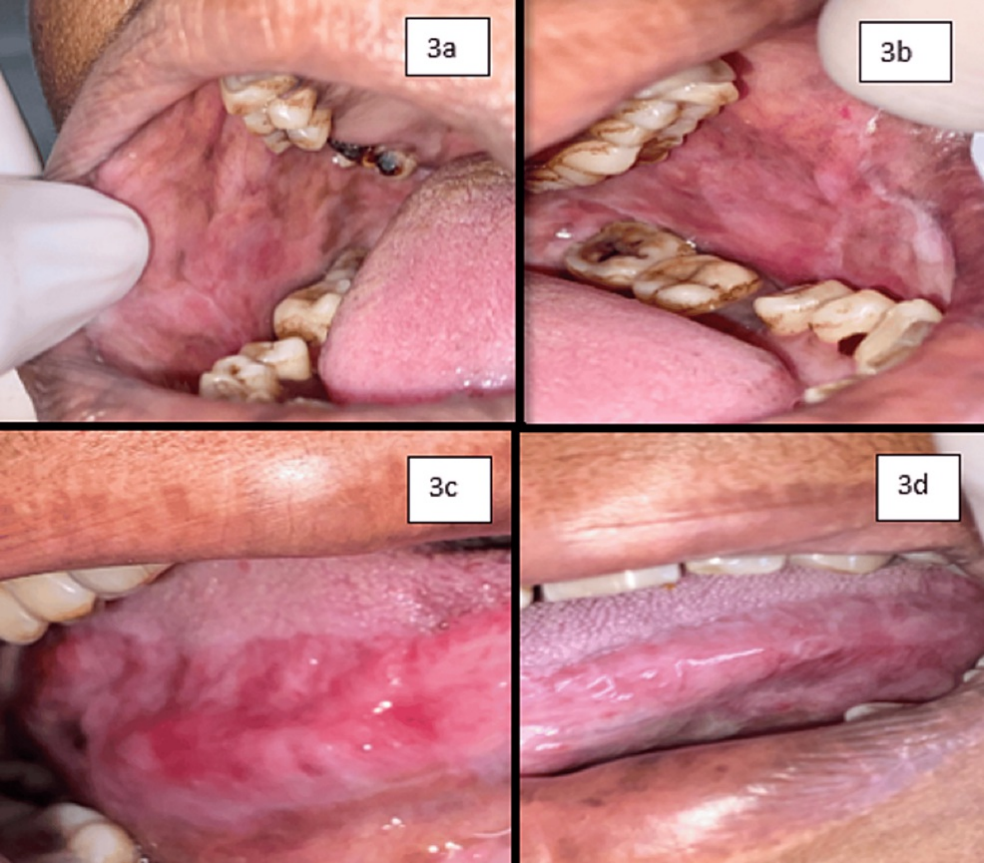
Evidence of the healing of lesions (90%-95%) on the third visit. Figure 3a shows the right buccal mucosa, Figure 3b shows the left buccal mucosa, Figure 3c shows the right lateral border of the tongue, and Figure 3d shows the left lateral border of the tongue.

On examination, inspectory findings revealed diffuse multiple ulcers on the right and left buccal mucosa extending toward the gingivo-buccal sulcus and retromolar region and also involving the lateral border of the tongue on both sides with sizes ranging from 0.3 to 1 cm and irregular shape, ill-defined margins with yellowish slough at the center, and lesion surrounded by erythematous area (Figure 1). Lesions on the right and left buccal mucosa and right lateral border of the tongue are continuous with each other, while ulcers on the left lateral border remain separated from one another. Tissue tags were also present alongside the lesion. There was tenderness with the lesion on all four sites as well. Nikolsky’s sign was not positive for this case. There were no similar lesions seen on the skin and in the genital area. Taking into consideration the history and clinical presentation, clinical diagnosis was given as pemphigus vulgaris, while erythema multiforme, erosive lichen planus, recurrent herpetic simplex infection, bullous pemphigoid, and mucous membrane pemphigoid were considered for differential diagnosis. As the patient was hypertensive, it was planned to treat the patient with local steroids. Thus, she was treated by prescribing 40 mg tablet prednisolone for swish and spit two times a day for three days along with 0.1% triamcinolone acetonide gel for local application four times a day. Fibronac tablet (aceclofenac 100 mg) was also given to reduce pain; then, the patient was recalled after three days. On the day of the first recall, oral examination showed significant improvement of the lesion with evidence of healing and reduced burning sensation (50%-60%) (Figure 2). The patient was asked to continue with the same medication for the next seven days. On the second recall, examination revealed a nearly completely healed ulcer (90%-95%) of the affected mucosa as shown in Figure 3. Regular follow-up of the patient was done where satisfactory results were achieved with no recurrence of the lesion.

## Discussion

PV is a mucocutaneous disease due to the autoimmune reaction of desmoglein by IgG autoantibody causing intra-epithelial acantholysis. Other ulcerative vesiculobullous lesions such as erythema multiforme, herpes simplex infection, bullous pemphigoid, and mucous membrane pemphigoid and premalignant lesion such as erosive lichen planus can be considered for differential diagnosis. If PV is left untreated, it may lead to fatal consequences with a mortality rate that ranges from 50% to 100% due to the extensive involvement of the mucosa and skin and the functional loss of epidermal barrier, which may lead to electrolyte imbalance along with systemic secondary bacterial infection. The initiation of early treatment and management could potentially prevent such serious complications [7,8].

Systemic corticosteroids along with topical steroids provide the first line of treatment to reduce inflammation in PV. Certain modifications apart from the conventional treatment may be required to manage PV in such patients and to avoid any adverse complications by avoiding the administration of systemic steroids and prescribing only topical application to the lesion as has been done in this case, in which the lesion responded very well to the medication [9].

If steroids are to be used on patients with systemic conditions, adjuvant medications such as azathioprine and mycophenolate compounds (immunosuppressants) can be used to prevent the side effects of steroids to a certain extent. At this time, rituximab along with corticosteroids is used as first-line treatment of moderate to severe pemphigus, which provide a corticosteroid-sparing effect to a large degree, thus decreasing the risk of steroid-associated adverse effects [10].

Other therapeutic options for the management of PV can be immunoadsorption where the removal of circulating antibodies can be achieved. This is mainly indicated when there is refractory PV where PV is resistant or nonresponsive to corticosteroids combined with azathioprine and mycophenolate. This treatment can be done on four consecutive days, and repeat the procedure after four weeks if required [11]. Therapeutic plasma exchange (plasmapheresis) has also been reported to be an effective adjuvant therapy for the management of PV that is severe. It can control the disease by reducing the serum level of autoantibody [12].

## Conclusions

In a patient suffering from PV and systemic conditions such as hypertension, it is important to curate the management meticulously especially when the standard drug of choice for the lesion can worsen the existing systemic disease, as in this case. It is important to modify the course of treatment to extract the benefit of the drug to the lesion and at the same time avoid its potential effect on the systemic condition of the patient. In this case presentation, the systemic administration of steroids was avoided. The patient was treated by topical application along with swish and spit of steroid where the patient did not consume the drug systemically. The local administration of these steroids provides significant improvement in the healing of the lesion without any systemic effects.
